# Defining the Erythrocyte Binding Domains of *Plasmodium vivax* Tryptophan Rich Antigen 33.5

**DOI:** 10.1371/journal.pone.0062829

**Published:** 2013-04-25

**Authors:** Hema Bora, Rupesh Kumar Tyagi, Yagya Dutta Sharma

**Affiliations:** Department of Biotechnology, All India Institute of Medical Sciences, New Delhi, India; Aligarh Muslim University, India

## Abstract

Tryptophan-rich antigens play important role in host-parasite interaction. One of the *Plasmodium vivax* tryptophan-rich antigens called PvTRAg33.5 had earlier been shown to be predominantly of alpha helical in nature with multidomain structure, induced immune responses in humans, binds to host erythrocytes, and its sequence is highly conserved in the parasite population. In the present study, we divided this protein into three different parts i.e. N-terminal (amino acid position 24–106), middle (amino acid position 107–192), and C-terminal region (amino acid position 185–275) and determined the erythrocyte binding activity of these fragments. This binding activity was retained by the middle and C-terminal fragments covering 107 to 275 amino acid region of the PvTRAg33.5 protein. Eight non-overlapping peptides covering this 107 to 275 amino acid region were then synthesized and tested for their erythrocyte binding activity to further define the binding domains. Only two peptides, peptide P4 (at 171–191 amino acid position) and peptide P8 (at 255–275 amino acid position), were found to contain the erythrocyte binding activity. Competition assay revealed that each peptide recognizes its own erythrocyte receptor. These two peptides were found to be located on two parallel helices at one end of the protein in the modelled structure and could be exposed on its surface to form a suitable site for protein-protein interaction. Natural antibodies present in the sera of the *P. vivax* exposed individuals or the polyclonal rabbit antibodies against this protein were able to inhibit the erythrocyte binding activity of PvTRAg33.5, its fragments, and these two synthetic peptides P4 and P8. Further studies on receptor-ligand interaction might lead to the development of the therapeutic reagent.

## Introduction

*Plasmodium vivax* is the commonest human malaria parasite. It has the widest distribution throughout the tropics, subtropics, and temperate zones [1]. Due to lack of continuous in vitro culture, characterization of *P. vivax* molecules has been very slow. As a result, only fewer *P. vivax* vaccine candidate antigens are under the clinical trials as compared to *P. falciparum*. Hence, there is a need to identify the newer *P. vivax* molecules which play important role in survival of the parasite inside its host, have very limited genetic diversity, and generate protective immune responses. Parasite molecules involved in host-parasite interaction play major role in the parasite’s life cycle. Molecules participating in this step may be exploited to design the therapeutic reagents which can inhibit the interaction of the parasite with its human host.

Tryptophan-rich antigens from *Plasmodium* species have been proposed as potential vaccine candidates. For the first time, tryptophan-rich antigens were characterized from *P. yoelii* where, pypAg1 and pypAg3 showed protective immune responses against infection in mice [2]. Immunization with recombinant pypAg1 reduced four to seven fold parasitemia against *P. yoelii* infection [3]. Subsequently, two such proteins termed as Tryptophan and Threonine-rich Antigen (TryThrA) and Merozoite associated Tryptophan-rich Antigen (MaTrA) were characterized from *P. falciparum*. These proteins had characteristics which were similar to *P. yoelii* antigens [4], [5]. Further studies on TryThrA led to the identification of peptides which could bind to normal human erythrocytes and also inhibited the *in vitro* merozoite invasion [6]. Later, Tryptophan-rich Antigen 3 (TrpA-3,) and Lysine-Tryptophan-rich Antigen (LysTrpA) of *P.falciparum* were characterized [7].

The genome sequencing of human malaria parasite *P.vivax* and its closest animal model represented by monkey malaria parasite *P.cynomolgi* revealed that each parasite has larger number of tryptophan-rich antigens [8], [9]. Characterization of these tryptophan-rich antigens is needed to develop the newer drug and vaccine targets. Earlier, we had identified the first *P. vivax* tryptophan-rich antigen and named it as PvTRAg [10]. It was followed by the characterization of many such proteins of this parasite which generated immune responses in *P. vivax* patients and did not show much genetic polymorphism in parasite population [10]–[17]. Six of 15 PvTRAgs, including PvTRAg33.5, have shown the erythrocyte binding activity [18]. Recently, we have reported the physico-chemical characterization and molecular modeling of PvTRAg33.5 [13]. This protein has also shown humoral and cellular immune responses in humans, and no genetic diversity in parasite population [19]. In the present study, we have defined the erythrocyte binding domains of PvTRAg33.5 and this binding activity was inhibited by the *P. vivax* patients’ sera.

## Materials and Methods

### Materials

For antibody inhibition assay, the heparinized blood (∼200 µl) was collected from the microscopically confirmed *P. vivax* malaria patients. Heparinized blood (2 ml) was also collected from the healthy lab individuals with B positive blood group for the erythrocyte binding assays. All individuals were informed about the study and their written consent was obtained for blood collection. Institutional ethical guidelines were followed during blood collection. Ethics committee of All India Institute of Medical Sciences, New Delhi, approved the study via approval number IEC/NP-342/2012 & RP-11/2012.

### Cloning, Expression and Purification of Three Fragments Derived from PvTRAg33.5

The cloning, expression, and purification of recombinant PvTRAg33.5 derived from exon-2 of *pvtrag33.5* gene have been described earlier [13]. Exon-2 of PvTRAg33.5 was further divided into three different parts i.e. N-terminal region (N-PvTRAg33.5 ) covering 70–318 bp (24–106 amino acid residues), middle region (M-PvTRAg33.5) covering 319–577 bp (107–192 amino acid residues) and C-terminal region (C-PvTRAg33.5) covering 555–828 bp (185–275 amino acid residues) by amplifying each region separately using *Pfx* polymerase and PvTRAg33.5-pGEM®T Easy recombinant clone as template. The N-terminal fragment was PCR amplified by using primers NTF 5′-TGTAGTCGACTCAAAGCGCAGTAG-3′ and NTR 5′- TCAAATATCTAGAAAAATTATTCC-3′ (Restriction sites engineered in primers are underlined). After Initial denaturation of template DNA at 94°C for 10 minutes, a total of 35 cycles were carried out under following conditions; denaturation at 94°C for 30 seconds, annealing at 50°C for 30 seconds, and extension at 68°C for 30 seconds. Final extension was at 68°C for 15 minutes. The other two fragments (middle and C-terminal) were amplified under the same conditions using primers MTF 5′-TCTGGATCCTTGAGTGATGGATAC-3′ and MTR 5′-TGAAGCCCAGTTTCTAGATTCCTG-3′ for M-PvTRAg33.5 and CTF 5′- GTTGGATCCATGTATTGGGAT-3′ and CTR 5′- TTGTTCCTAATTGAGTCTAGAATTCC-3′ (Restriction sites engineered in primers are underlined) for C-PvTRAg33.5. The PCR products were cloned in to the pPROEX^TM^HT expression vectors and Histidine-tagged protein was purified using immobilized metal affinity chromatography on Ni^2+^ NTA agarose column according to manufacturer’s instructions (Qiagen, GmbH, Hilden, Germany). Since C-PvTRAg33.5 could not be purified, it was recloned in pGEX4T-2 vector, which adds GST-tag at the N-terminal region of the protein, and purified using Glutathione Sepharose™ 4B resin as per manufacturer’s instructions (GE Healthcare Bio-Sciences AB, Uppsala, Sweden). The homogeneity of purified recombinant proteins was confirmed by SDS-PAGE as described earlier [12].

### Erythrocyte Binding Assay by Cell – ELISA

Erythrocyte binding activity of tagged PvTRAg33.5 and its fragments was analyzed as described elsewhere [20]. Briefly, each well of a 96-well microtiter plate was coated with one million human erythrocytes of B positive blood group individuals and incubated overnight at 4°C. After washing with PBS and blocking with 5% BSA for 2 h at 37°C, different concentrations (0.015, 0.03, 0.062, 0.125, 0.25, 0.5, 1, and 2 µM) of purified Histidine-tagged PvTRAg33.5, N-PvTRAg33.5, M-PvTRAg33.5, or GST tagged C-PvTRAg33.5 were added. The plates were incubated for 4 h at room temperature. After washing, the plates were developed with anti-His_6_ or anti-GST (for C-PvTRAg33.5) monoclonal antibodies (Sigma Aldrich, St. Louis, USA) followed by horseradish peroxidase (HRP) conjugated anti-mouse IgG secondary antibody (Pierce Biotechnology Inc., Rockford, IL, USA) and o-phenyldiamine (OPD) substrate (Sigma-Aldrich, St. Louis, MO, USA). Optical density was recorded at 490 nm. A 23 kDa Histidine-tagged thioredoxin from *Desulfovibrio desulfuricans* [21] and recombinant GST protein were was used as negative controls. The absorbance of GST alone was subtracted from GST-tagged C-PvTRAg33.5 in binding experiments.

For antibody inhibition assay, the tagged PvTRAg33.5, its fragments or peptides (250 nM) were incubated overnight at 4°C with various dilutions (1∶10, 1∶50, 1∶100, and 1∶1000) of rabbit anti-PvTRAg33.5 antibodies and then added to erythrocytes in a micro titer plate. Plates were then developed with anti-His_6_ or anti-GST monoclonal antibody as described above. Same protocol was followed with pooled *P. vivax* infected patient sera at dilutions 1∶10, 1∶30, 1∶50, 1∶75 and 1∶100. The anti-PvTRAg33.5 antibodies were also affinity purified from the pooled *P. vivax* infected patient sera using CarboxyLink™ Immobilization Kit (Thermo scientific, Rockford, USA) according to manufacturer’s protocol. Briefly, 2 mg of PvTRAg33.5 was coupled to the resin. After washing with PBS, bound antibody was eluted by 0.2 M Glycine-HCl buffer. The purified antibody was dialyzed in 50 mM Tris-HCl, diluted to the same level as of the initial serum, and used in the erythrocyte binding inhibition assay.

### Peptide Designing

In order to further define the erythrocyte binding region of PvTRAg33.5, non-overlapping peptides were commercially synthesized (Thermo fisher Scientific, GmbH, Germany). A stretch of six histidines was added to the C-terminal end of each peptide so that the peptide bound to erythrocytes could be detected by anti-His_6_ monoclonal antibody. Erythrocyte binding activity of these peptides was assessed by Cell-ELISA, as above. The inhibition of erythrocyte binding activity of peptides by antibodies was also checked by Cell-ELISA. Experiments were performed in duplicate wells and repeated at least three times.

### Competition Assay

As mentioned above, approximately one million erythrocytes coated in to each well of the 96 well microtiter plate and blocked with 5% BSA was incubated with 5 µM of peptide P4 or P8 or P4 plus P8, or 2 µM of Histidine tagged complete PvTRAg33.5 for 3 h at room temperature. After washing, 1 µM of GST-tagged complete PvTRAg33.5 was added to each well and plate was incubated for 3 h at room temperature. After washing, the plates were incubated with monoclonal antibodies against GST and processed further as described above. For (no competition) control, erythrocytes were directly incubated with GST- tagged complete PvTRAg33.5 or GST alone and plate was developed as above. The absorbance obtained for GST alone was subtracted from readings obtained for the GST- tagged protein.

## Results

### Defining the Erythrocyte Binding Regions of PvTRAg33.5

**Figure 1:**
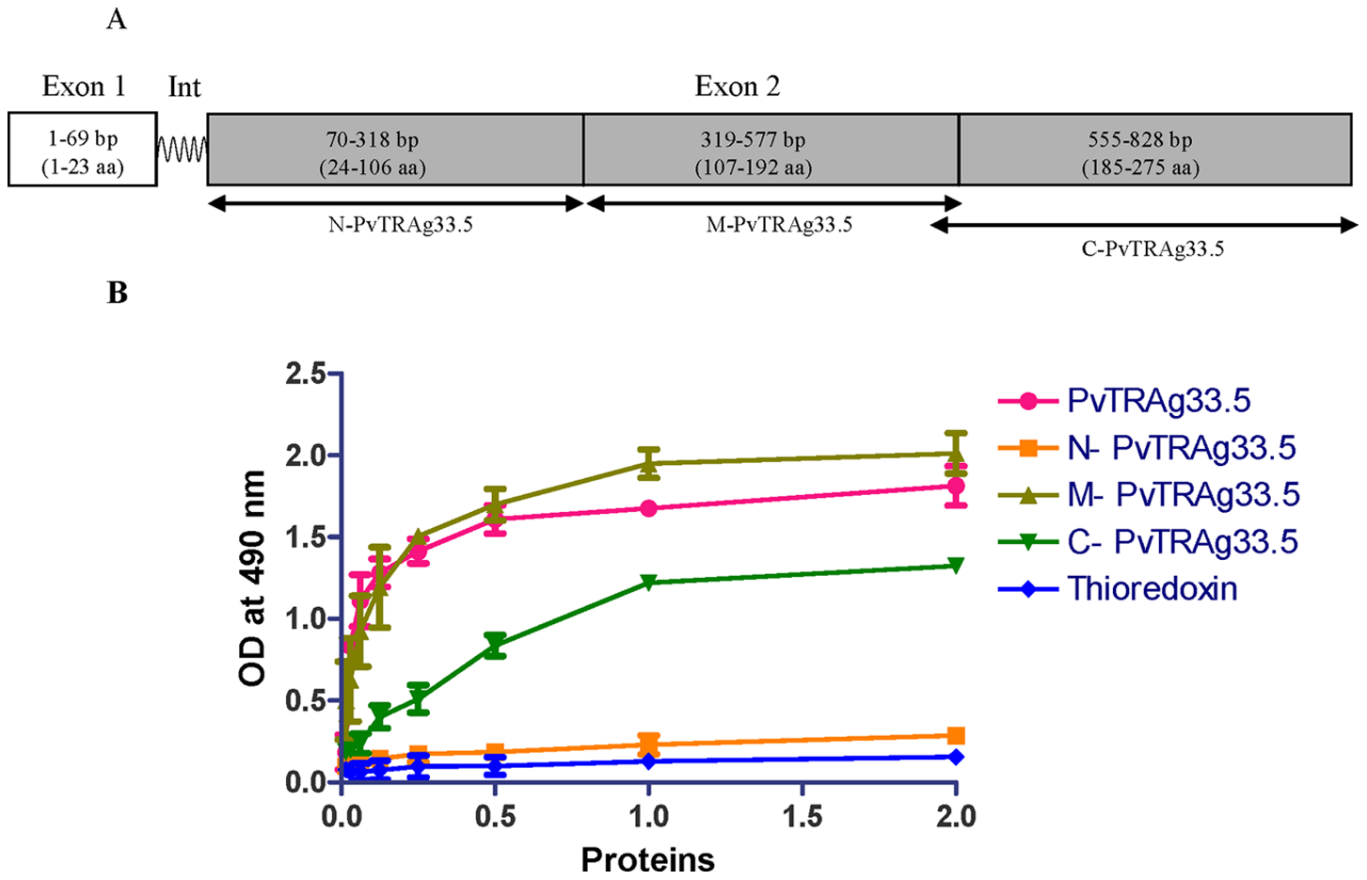
Determination of erythrocyte binding regions of PvTRAg33.5. (**A**) Schematic representation of PvTRAg33.5. Exon 1 encodes for a 23 amino acid signal peptide. Wavy lines indicate the intron. Exon 2 (shaded grey) encodes the mature protein which was fragmented in to three parts i.e. N-PvTRAg33.5, M-PvTRAg33.5 and C-PvTRAg33.5. (**B**) Cell-ELISA showing erythrocyte binding of Histidine-tagged PvTRAg33.5 and its three fragments with human erythrocytes (C-PvTRAg33.5 was GST-tagged). Increasing concentrations of the purified recombinant proteins were allowed to bind with ∼1 million erythrocytes in a microtiter plate and reacted with primary anti-His_6_ or anti GST antibody and HRP conjugated secondary antibody. Recombinant Histidine-tagged thioredoxin from *D. desulfuricans* was used as negative control. Error bar indicates the standard deviation of mean from three experiments. Int, intron.

As shown in figure **(** **Fig.** **1A** **),** the PvTRAg33.5 was divided into three parts, named as N-terminal (N-PvTRAg33.5), middle (M-PvTRAg33.5) and C-terminal (C-PvTRAg33.5) fragments. These fragments were expressed in *E.coli*, and purified recombinant peptides containing either the Histidine-tag or GST-tag were used for erythrocyte binding assay. There was an overlap of 8 amino acids between M-PvTRAg33.5 and C-PvTRAg33.5 fragments. Results showed that the middle (M-PvTRAg33.5) and C- terminal (C-PvTRAg33.5) fragments bind to human erythrocytes in a concentration dependent manner (**Fig. 1B**). The N-terminal (N-PvTRAg33.5) fragment comprising of initial 106 amino acids did not show binding to the human RBC.

### Further Defining of the Erythrocyte Binding Domains of PvTRAg33.5

**Figure 2:**
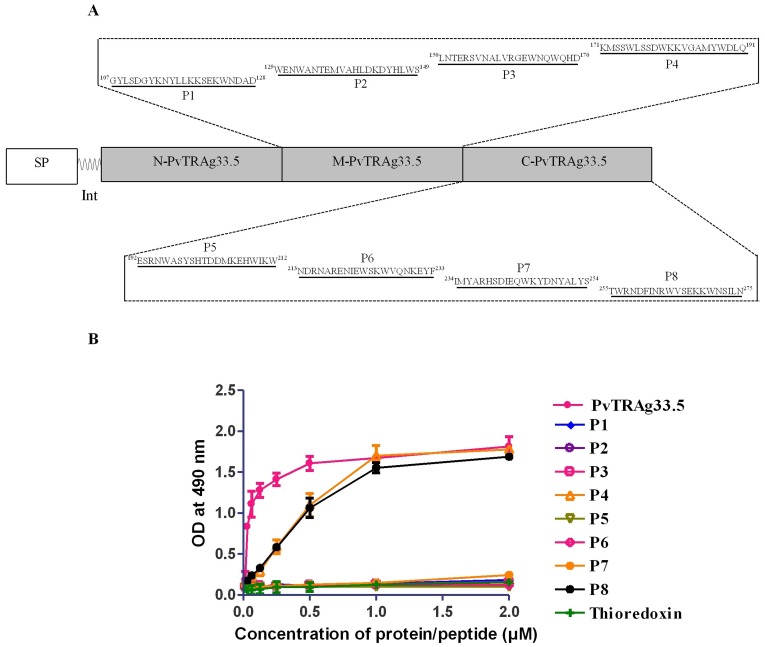
Determination of erythrocyte peptide binding domains of PvTRAg33.5. (**A**) Schematic representation of eight non-overlapping peptides designed from M-PvTRAg33.5 and C-PvTRAg33.5 fragments. Amino acid sequence and name of each peptide along with residue numbers is shown. (**B**) Cell-ELISA showing erythrocyte binding affinity of these synthetic non overlapping peptides. Increasing concentrations of these peptides were allowed to bind with erythrocytes. Reaction with primary and secondary antibodies was carried out as described in Fig. 1B. Recombinant thioredoxin from *D. desulfuricans* was used as negative controls. Mean± SD value of absorbance from three experiments is plotted. SP, signal peptide; Int, intron.

Since middle and C- terminal fragments of PvTRAg33.5 bind to human erythrocytes, a total of eight different non-overlapping peptides (each peptide of 21 amino acid length except peptide P1 which was 22 amino acid long) were synthesized from this region (107 to 275 amino acid position) of the protein (**Fig. 2A**). These peptides were then tested for their binding activity to the human erythrocytes. The peptide P4 (KMSSWLSSDWKKVGAMYWDLQ) located at 171–191 amino acid position (represented in M-PvTRAg33.5 fragment) and peptide P8 (TWRNDFINRWVSEKKWNSILN) at 255–275 amino acid position (represented in C-PvTRAg33.5 fragment), showed binding activity towards the uninfected human erythrocytes in a concentration dependent manner **(** **Fig. 2B** **)**. Rest of the peptides (P1, P2, P3, P5, P6, and P7) did not bind to these erythrocytes. Complete recombinant PvTRAg33.5 protein and bacterial thioredoxin were used as positive and negative controls, respectively.

### Natural Antibodies Inhibit Erythrocyte Binding Activity of PvTRAg33.5 Fragments and its Peptides

**Figure 3:**
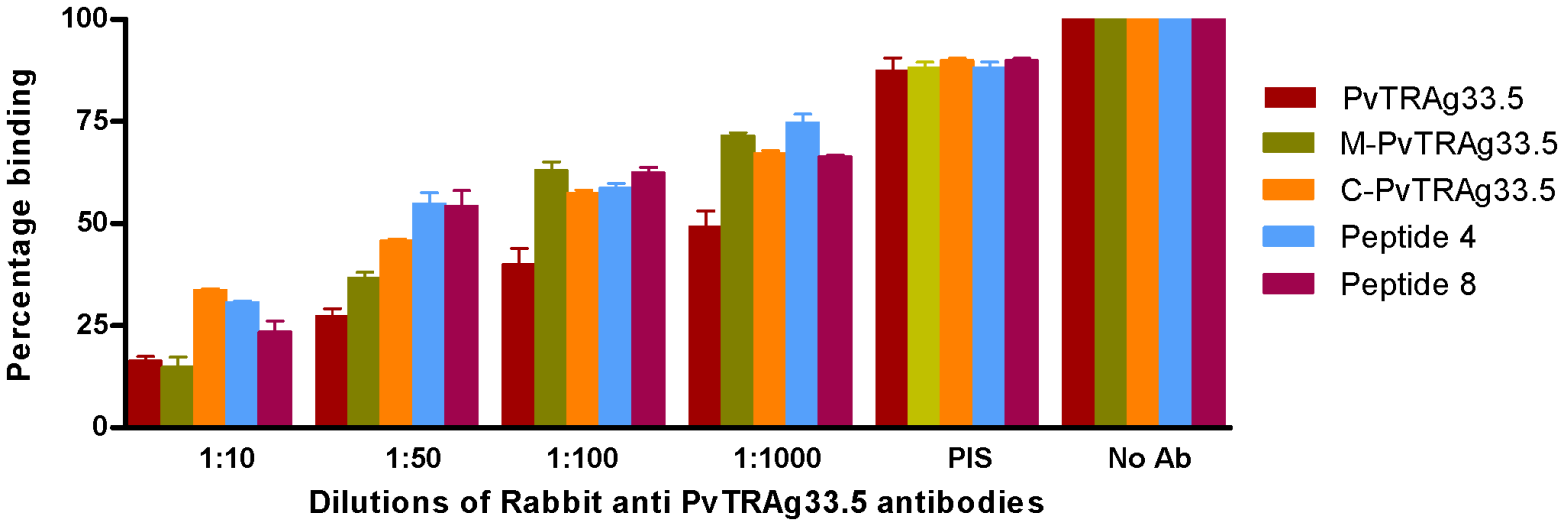
Inhibition of erythrocyte binding of PvTRAg33.5 derived fragments and peptides by rabbit anti-PvTRAg33.5 antibody. The tagged recombinant PvTRAg33.5, its fragments, or synthetic peptides were mixed with different dilutions of polyclonal antisera raised in rabbit against PvTRAg33.5 before adding to the microtiter plate coated with erythrocytes. Further steps of color development were same as in Fig. 1 and 2B. Binding in the absence of antibody was taken as percentage control. Error bar indicates the standard deviation of mean from three experiments. No Ab, no antibody; PIS, pre-immune rabbit sera.

**Figure 4:**
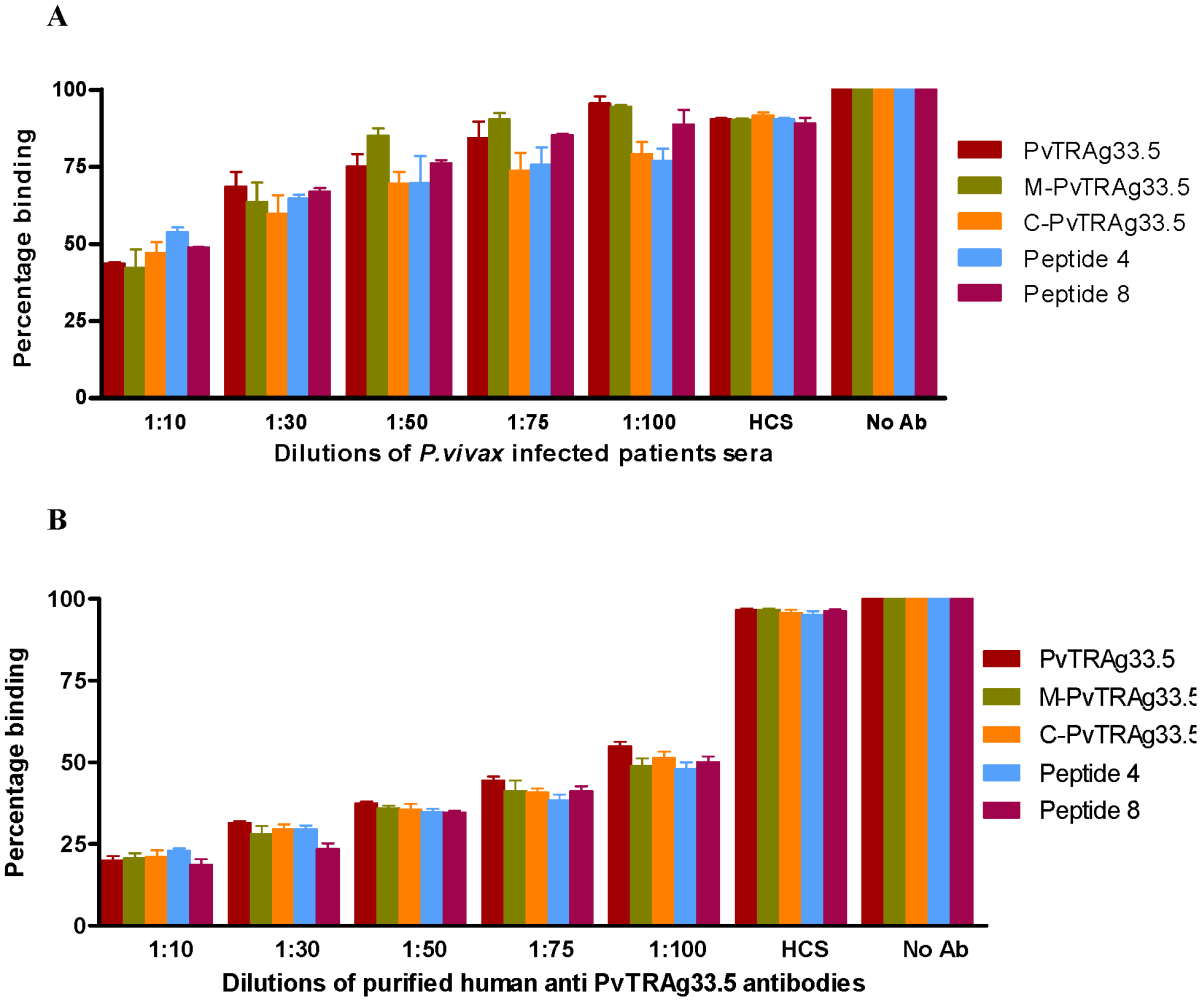
Inhibition of erythrocyte binding of PvTRAg33.5 derived fragments and peptides by *P. vivax* patients’ sera. Pooled *P. vivax* infected patients’ sera (**A**) and by purified anti-PvTRAg33.5 antibodies from these pooled patients sera (**B**) were used for these antibody mediated inhibition assays. The tagged recombinant PvTRAg33.5, its fragments, or synthetic peptides were mixed with different dilutions of *P.vivax* patients’ sera or purified antibodies before adding to the microtiter plate coated with one million erythrocytes. Further steps of color development were same as in Fig. 1 and 2B. Binding in the absence of antibody is taken as percentage control. Error bar indicates the standard deviation of mean from three experiments. No Ab, no antibody; HCS, healthy human control sera.

The M-PvTRAg33.5, C-PvTRAg33.5, and peptides P4 and P8 were incubated separately with various dilutions of Rabbit anti-PvTRAg33.5 antibody before allowing their binding to uninfected erythrocytes. Results showed that this antibody inhibited binding of these recombinant or synthetic peptides to human erythrocytes in a dilution dependent manner (**Fig. 3**). Similarly, natural antibodies present in the *P. vivax* patients’ sera also inhibited their binding to these erythrocytes **(** **Fig. 4** **)**. Pre-immune rabbit sera or sera from healthy uninfected individuals did not affect the binding. In both the experiments, shown in figures 3 and 4, it was observed that the rabbit sera inhibited the erythrocyte binding at a higher dilution as compared with the patients’ sera. This could arise if the rabbit sera had higher titers of antibodies than the patients’ sera or patient sera had some non-specific antibodies. For this purpose, the anti-PvTRAg33.5 antibodies were affinity purified from the pooled *P.vivax* patients’ sera and used for inhibition assay. It was observed that purified anti-PvTRAg33.5 antibodies from patients’ sera inhibited the erythrocyte binding activity of these recombinant and synthetic peptides more efficiently than the unpurified sera **(** **Fig. 4B** **)**. This difference in binding inhibition by the purified antibody was statistically significant (P<0.05).

### Peptide P4 and P8 Recognize their own Erythrocyte Receptor

**Figure 5:**
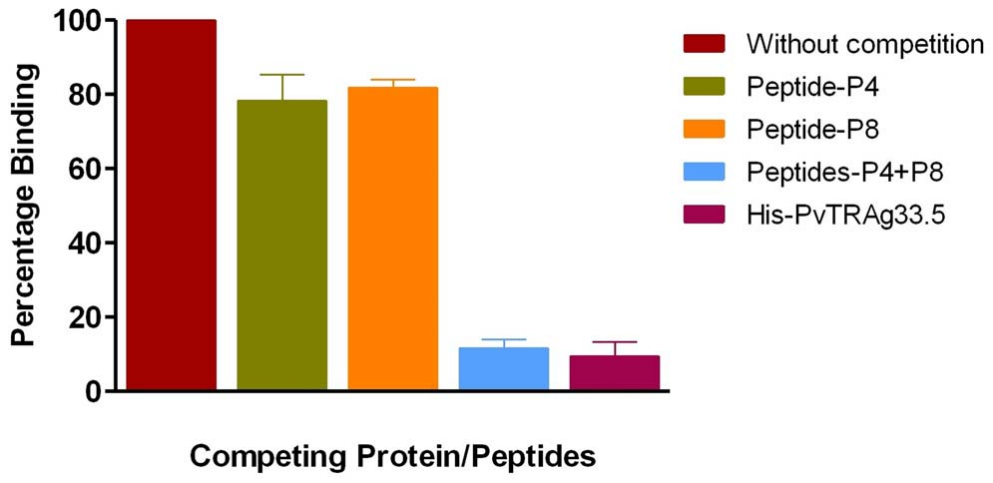
Competition of peptide P4 and P8 with complete PvTRAg33.5 in erythrocyte binding assay. Approximately one million erythrocytes in each well of a 96 well microtiter plate were incubated with 5 µM of peptides or 2 µM of Histidine tagged complete PvTRAg33.5 protein. In a control well, no protein or peptide was added to the erythrocytes. The plate was then incubated with 1 µM of GST- tagged complete PvTRAg33.5, and developed with anti GST monoclonal antibodies, as described in Fig. 1B. Error bar indicates the standard deviation of mean from three different experiments.

Erythrocyte binding of peptide P4 and P8 has been established above. Each of these two peptides was then allowed to compete with complete recombinant (GST-tagged) PvTRAg33.5 protein in erythrocyte binding assay by Cell-ELISA. Results showed a partial inhibition in binding activity of complete PvTRAg33.5 to RBC, if each peptide was allowed to compete with it separately. But the combined mixture of P4 and P8 peptides was able to inhibit the binding of the complete PvTRAg33.5 to RBC almost completely (**Fig. 5**). This complete inhibition of erythrocyte binding by the mixture of these two peptides was similar to that of the control where competition was allowed between recombinant Histidine-tagged PvTRAg33.5 and the GST- tagged PvTRAg33.5 complete proteins (**Fig. 5**). These results therefore suggest that each peptide recognizes its own receptor on human erythrocyte.

## Discussion

Tryptophan-rich proteins of malarial parasites play important role in parasite survival, as reported in case of murine malaria caused by *P. yoelii* [2], [3], [22]. *Plasmodium vivax* contains the largest number of such proteins and earlier we have been able to characterize some of them, including PvTRAg33.5 [10]–[19]. Here, we have characterized this protein further and shown that it binds to the uninfected host erythrocytes through two different peptide regions which come closer on the surface of the protein in its 3D structure, and this binding is inhibited by the patients’ sera.

**Figure 6:**
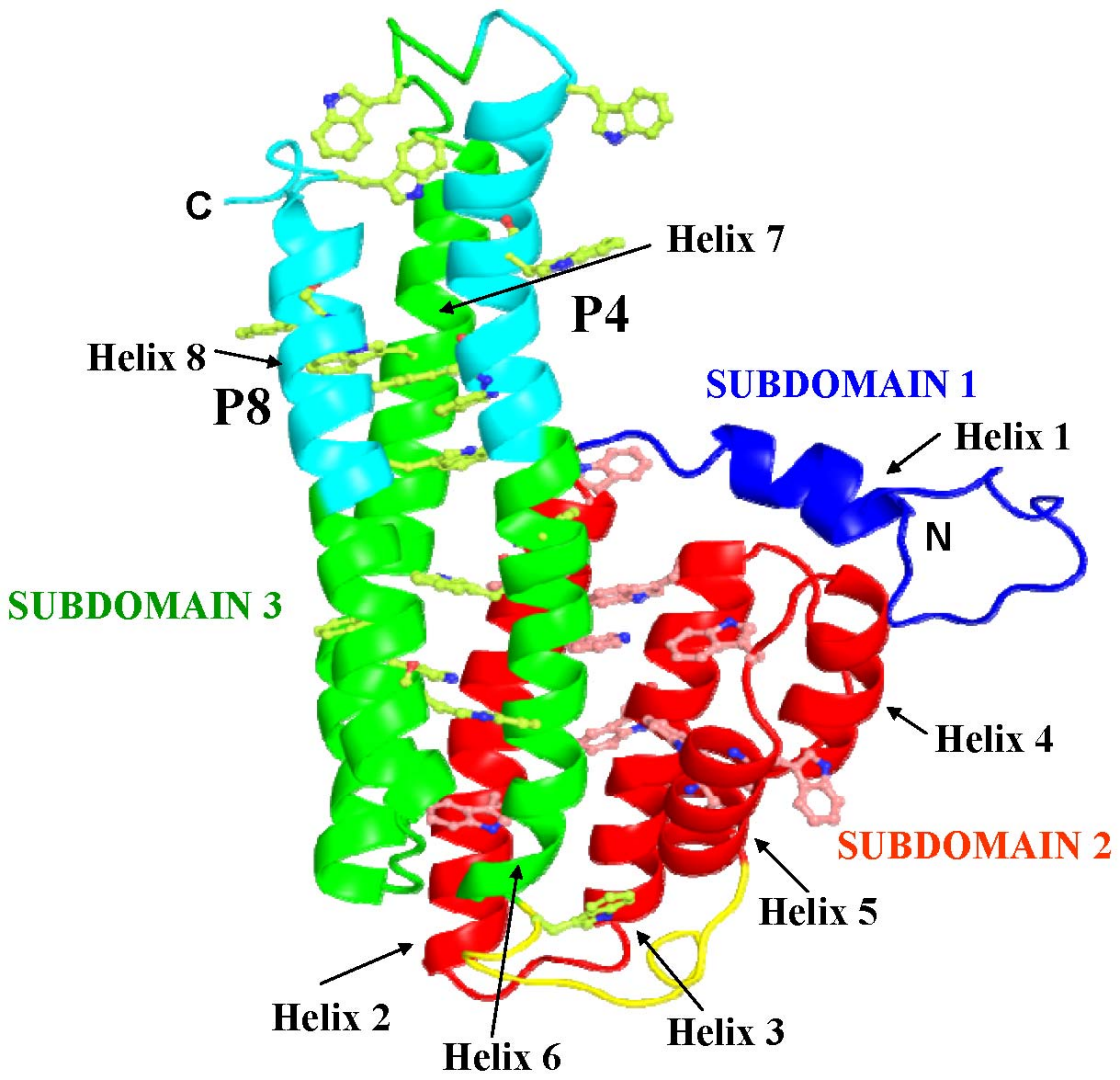
Location of erythrocyte binding peptide domains on modeled PvTRAg33.5. PvTRAg33.5 has three subdomains. Subdomain 1 (blue), Subdomain 2 (red), and subdomain 3 (green). The helices of different subdomains are shown in respective colors and coiled structure as loops. Erythrocyte binding peptides P4 (in helix 6) and P8 (in helix 8) are shown in cyan color.

Previous studies have shown that tryptophan-rich antigens of *Plasmodium* species interact with uninfected host erythrocytes [6], [12], [22]. Since we have already shown that PvTRAg33.5 binds to the host erythrocytes [18], we wanted to define the binding domains of this protein further. Hence, PvTRAg33.5 was divided in to three different parts i.e. N-terminal region (amino acid position 24–106), middle (amino acid position 107–192), and C-terminal region (amino acid position 185–275) and tested them for their erythrocyte binding activity. Middle and C-terminal fragments showed the erythrocyte binding activity (**Fig. 1**). Further exclusion of non-binding region came from the synthetic peptide binding studies where peptide P4 (amino acid position 171–191) and peptide P8 (amino acid position 255–275) showed erythrocyte binding. Thus we were able to define two peptide domains in PvTRAg33.5 which were 63 amino acids apart and showed specific binding to host RBCs (**Fig. 2B**). These two peptides P4 and P8 were localized in the modeled tertiary structure of PvTRAg33.5 [13] where peptide P4 was found to be located towards the end of the helix 6 as well as the beginning of the coil joining helix 6 and 7 whereas peptide P8 is located towards the posterior region of helix 8 as well as the C terminal loop (**Fig. 6**). Both these peptides are located in subdomain 3 of the protein on two parallel helices. These peptide sequences may be exposed to the surface of the protein to form a suitable binding site for interaction.

Enzymatic treatments to remove specific surface receptors have helped in the identification of molecules which interact with the parasite proteins and mediate the crucial step of host cell invasion by the parasite [23]–[28]. During our earlier studies, we had ruled out the involvement of glycophorins, sialglycoproteins and band-3 protein of erythrocytes as PvTRAg33.5 receptor(s). This is because the erythrocytes treated with trypsin, chymotrypsin and neuraminidase did not show any effect on their binding affinity towards PvTRAg33.5 [18]. This is similar to PfRH5 (Pf reticulocyte homolog 5) where binding occurs with a molecule which is neither sialoglycoprotein nor band-3 [29], [30]. Further studies are required to identify RBC receptors for this protein. However, cross-competition of PvTRAg33.5 with other RBC binding PvTRAgs revealed that it has two erythrocyte receptors. One of its RBC receptor is shared by PvTRAg38 and other is shared by PvATRAg74. On the other hand, both of its erythrocyte receptors were shared by PvTRAg35.2, PvTRAg69.4, and PvTRAg [18]. Since PvTRAg33.5 has two erythrocyte binding domains (P4 and P8), it is possible that each peptide domain recognizes its own RBC receptor. This was proven by the competition assay where each peptide was able to partially compete with PvTRAg33.5 during erythrocyte binding. However, in combination (peptides P4 and P8 together) they completely inhibited the PvTRAg33.5 binding to erythrocytes (**Fig. 5**). This suggests that each peptide recognizes its own RBC receptor but both domains are required for efficient binding. This receptor-ligand interaction between PvTRAg33.5 (or its peptides) and erythrocyte protein is blocked by the PvTRAg33.5 antibodies whether raised in rabbit or purified from the *P. vivax* patients’ sera (**Fig. 3** **,** **4**).

Earlier, we have shown that the PvTRAg33.5 sequence is highly conserved in the *P. vivax* parasite population [19]. Therefore, the erythrocyte binding domains (P4 and P8 sequences) will also remain conserved among field isolates. As mentioned above, the natural antibodies could block this peptide binding to its host erythrocyte. This information on host-parasite interaction then could be utilized to develop the immunotherapeutic reagents. These peptides thus can form the basis of a minimal subunit based, multiepitopic, multistage anti malarial vaccine. An alternative approach which utilizes the chemical synthesis of peptides that resemble key regions of proteins on the pathogen’s surface or have role in host-parasite interaction can also be deployed to develop the therapeutic agents [31]. This approach has been used for the peptides derived from various *P. falciparum* proteins showing binding affinity to host erythrocytes and their role in RBC invasion [6], [32]–[37].

In conclusion, two domains at amino acid position 171–191 (P4) and 255–275 (P8) of PvTRAg33.5 bind to host erythrocyte where each domain recognizes its own erythrocyte receptor. This binding is inhibited by polyclonal sera raised against PvTRAg33.5 as well as by the natural antibodies produced during *P. vivax* infection. Further studies are required to identify its RBC receptor which can lead to the development of a therapeutic reagent.
